# The Involvement of Ca_V_1.3 Channels in Prolonged Root Reflexes and Its Potential as a Therapeutic Target in Spinal Cord Injury

**DOI:** 10.3389/fncir.2021.642111

**Published:** 2021-03-23

**Authors:** Mingchen C. Jiang, Derin V. Birch, Charles J. Heckman, Vicki M. Tysseling

**Affiliations:** ^1^Department of Physiology, Feinberg School of Medicine, Northwestern University, Chicago, IL, United States; ^2^Physical Therapy and Human Movement Sciences, Feinberg School of Medicine, Northwestern University, Chicago, IL, United States; ^3^Physical Medicine and Rehabilitation, Feinberg School of Medicine, Northwestern University, Chicago, IL, United States

**Keywords:** spinal cord injury, muscle spasm, root reflex, Ca_V_1.3 channel, motoneuron

## Abstract

Spinal cord injury (SCI) results in not only the loss of voluntary muscle control, but also in the presence of involuntary movement or spasms. These spasms post-SCI involve hyperexcitability in the spinal motor system. Hyperactive motor commands post SCI result from enhanced excitatory postsynaptic potentials (EPSPs) and persistent inward currents in voltage-gated L-type calcium channels (LTCCs), which are reflected in evoked root reflexes with different timings. To further understand the contributions of these cellular mechanisms and to explore the involvement of LTCC subtypes in SCI-induced hyperexcitability, we measured root reflexes with ventral root recordings and motoneuron activities with intracellular recordings in an *in vitro* preparation using a mouse model of chronic SCI (cSCI). Specifically, we explored the effects of 1-(3-chlorophenethyl)-3-cyclopentylpyrimidine-2,4,6-(1H,3H,5H)-trione (CPT), a selective negative allosteric modulator of Ca_V_1.3 LTCCs. Our results suggest a hyperexcitability in the spinal motor system in these SCI mice. Bath application of CPT displayed slow onset but dose-dependent inhibition of the root reflexes with the strongest effect on LLRs. However, the inhibitory effect of CPT is less potent in cSCI mice than in acute SCI (aSCI) mice, suggesting changes either in composition of Ca_V_1.3 or other cellular mechanisms in cSCI mice. For intracellular recordings, the intrinsic plateau potentials, was observed in more motoneurons in cSCI mice than in aSCI mice. CPT inhibited the plateau potentials and reduced motoneuron firings evoked by intracellular current injection. These results suggest that the LLR is an important target and that CPT has potential in the therapy of SCI-induced muscle spasms.

## Introduction

Spinal cord injury (SCI) is often associated with paralysis or the lack of volitional movement, but up to 80% of people with SCI also develop involuntary muscle contractions, including spasms, which can severely impact function and safety (Maynard et al., 1990; Johnson et al., 1998; Noreau et al., 2000). Baclofen, the most widely used medication for spasms, works by binding to GABAB receptors to inhibit monosynaptic and polysynaptic spinal reflexes (Kita and Goodkin, 2000). Benzodiazepines, which target GABAA receptors, are also prescribed to reduce spasms (Kita and Goodkin, 2000). However, the clinical applications of these medicines are limited by their considerable side effects, which include but are not limited to sedation, nausea, dizziness and even difficulty in breathing (Kita and Goodkin, 2000; Dario and Tomei, 2004). In addition, benzodiazepines may have an additive effect when used in combination with baclofen (Kita and Goodkin, 2000). Thus, the search for better and more accurately targeted therapies with fewer side effects is necessary.

Although many mechanisms underlying spasms are still unknown, enough has been uncovered to begin looking at targets for therapies. The physiological basis for spasms can be divided into two portions: increased excitatory and decreased inhibitory inputs to the motoneuron (MN) and hyperexcitable MNs (Murray et al., 2011b). The increased synaptic excitation is derived from many different sources. Interneuronal populations have been implicated in being a part of the motoneuronal input and include V3 interneurons, glutamate-positive excitatory interneurons, and bursting deep dorsal horn interneurons (Thaweerattanasinp et al., 2016, 2020; Bellardita et al., 2017; Lin et al., 2019; Marcantoni et al., 2020). Other inputs include the loss of descending inhibition of norepinephrine and serotonin on sensory input (Murray et al., 2011b; Rank et al., 2011), reduction of inhibitory postsynaptic potentials (IPSPs) (Norton et al., 2008) and up-regulation of NMDA receptors on spinal MNs (Tu et al., 2017). All of these inputs may enhance mono- and polysynaptic excitatory postsynaptic potentials (EPSPs) to MNs. However, they summate on MNs that themselves have changes, including a clear resurgence in persistent inward currents (PICs) that amplify those signals and in turn, produce a prolonged response. Both sodium and calcium inward persistent currents (Na-, Ca-PICs) were found to be enhanced in SCI rats (Westenbroek et al., 1998; Li and Bennett, 2003; Li et al., 2004). Although the SCI related spasms can result from several pathophysiological changes in both synaptic and intrinsic properties in the spinal motor system, targeting the MN, which is the final output for this activity, would allow for better control of the involuntary motor behavior. Indeed, among several types of calcium channels (Westenbroek et al., 1998), Cav1.3, one subtype of L-type calcium channels (LTCCs), has been identified as the channel underlying PIC activity in motoneurons and spasms in SCI rats (Li and Bennett, 2003). Targeting intrinsic motoneuronal properties with nimodipine, an inhibitor of LTCCs, has been suggested as a potential therapy for SCI related spasms (Li and Bennett, 2003; Li et al., 2004). Recently, nimodipine was reported to inhibit the development of spasms in a mouse model of SCI (Marcantoni et al., 2020). These observations suggest that the LTCCs, i.e., Ca_V_1.2 and Ca_V_1.3, play an important role in SCI spasms. However, the application of nimodipine could be limited by its potential side effects in cardiovascular system (Lipscombe et al., 2004) because it has poor selectivity between Ca_V_1.2 and Ca_V_1.3 as Xu and Lipscombe (Xu and Lipscombe, 2001) reported that at 1 μM, nimodipine blocked 90% Ca_V_1.2-conducted current and 50% Ca_V_1.3 conducted current, and because Ca_V_1.2 channels are abundant in cardiovascular systems (Lipscombe et al., 2004). Recently, 1-(3-chlorophenethyl)-3-cyclopentylpyrimidine-2,4,6-(1H,3H,5H)-trione (CPT) has been reported to produce inhibitory effect on Ca_V_1.3 channels with high selectivity (Kang et al., 2012; Xie et al., 2016), and is known now as a selective negative allosteric modulator, i.e., inhibitor, for Ca_V_1.3 (Cooper et al., 2020). Thus, CPT could be an effective and safe therapy for muscle spasms after SCI.

This study used a mouse model of SCI due to its advantages, which include low cost, easy maintenance and tremendous genetic potentials (Rosenthal and Brown, 2007; Bryda, 2013). Only female nice were chosen because it is easier to express the bladders of female SCI mice and they have lower risk of complication (Lilley et al., 2020). In addition, the study with only female mice are unlikely to cause experimental bias because the pathophysiology in male and female SCI animals appears to be similar (Walker et al., 2019). The feasibility of the mouse model for this study is based on the facts that MNs in the mouse spinal cord are known to contain PICs (Meehan et al., 2010), and spasms and enhanced spinal cord reflex have been well demonstrated in the SCI models with injury at T9-10 (Skinner et al., 1996; Corleto et al., 2015; Tysseling et al., 2017; Mekhael et al., 2019). The MN portion of the spasm is represented by prolonged root reflexes, i.e., long lasting reflexes (LLRs), which last 2–10 s in ventral roots following a mild stimulation (Li et al., 2004; Murray et al., 2011a). In this study, we measured both ventral root reflexes and intrinsic firings in MNs in the sacral spinal cord to evaluate the effectiveness of CPT in the mouse model. Our results suggest a hyperexcitability in the spinal motor system in these SCI mice and support the idea that CPT has therapeutic potential for spasms in persons with SCI.

## Materials and Methods

### Experimental Animals

In this study, adult C57BL6/J female mice of 8 weeks were acquired from the Jackson Laboratory and housed under constant temperature (21 ± 2°C) in the animal facility of Northwestern University. All experimental procedures were reviewed and approved by the University Animal Research Committee and were in accordance with the Public Health Service Policy on Humane Care and Use of Laboratory Animals.

### Mouse Spinal Cord Injuries and Animal Care

SCI surgery was performed when the mice were 10 weeks of age. Mice were anesthetized using inhalation anesthetic (2.5% isofluorane in 100% oxygen administered using a VetEquip Rodent anesthesia machine). A laminectomy was performed at the T10 vertebral segment followed by a complete transection of the spinal cord with a Vannas spring scissors (F.S.T. 15002-08). Afterward, the skin was sutured using Autoclip (9 mm; BD Biosciences, San Jose, CA). Mice that exhibited any hindlimb movement 24 h after the injury were excluded from the study. These mice were allowed to age for an additional 10 weeks before the *in vitro* experiments in order to produce a chronic SCI mouse model. This model is distinguished from control mice with acute spinal cord transections (aSCI). Bladders were manually expressed twice daily. Meloxicam (0.3 mg/kg, s.c. q.d.) was administered once daily for the first 2 days, and additional doses were administered whenever mice showed signs of discomfort such as decreased activity and movement, self-chewing, excessive grooming, or anorexia (Jellish et al., 2008; Kazemi et al., 2015). Gentamycin (20 mg/kg s.c., q.d. for 5 days) was planned to treat urinary tract infections, but they did not occur in this study. Locomotor recovery after SCI was assessed using the Basso, Beattie, and Bresnahan (BBB) scale adapted for mice and the Basso Mouse Scale (BMS) (Joshi and Fehlings, 2002). In cSCI mice, muscle spasms in hindlimbs due to surgery handling could be observed in each experiment before anesthesia, which is similarly observed in other laboratory (Lin et al., 2019) and suggests the animals are ready for the experiment.

### Sacral Cord Preparation

Ventral root reflexes and intracellular MN activities were recorded from the sacral cords of both aSCI and cSCI mice. Ventral roots at sacral segments 2–3 (S2–S3) of the spinal cord were chosen to record root reflexes because they are caudal to the injury and are well characterized electrophysiologically (Jiang et al., 2015). The surgical procedure was adopted from previous publications (Jiang et al., 2009, 2017). In detail, mice were deeply anesthetized with intraperitoneal injections of urethane at 0.18 g/100 g and supplemental anesthesia (0.01–0.05 g/100 g) was determined by the mouse’s response to foot pinching with forceps. The spinal cord was exposed from T10 to the end of the sacral segment and then superfused with artificial cerebrospinal fluid (ACSF) at a flow rate of 5–7 ml/min. The ASCF was composed of the following (in mM): 120 NaCl, 3 KCl, 1 NaH_2_PO_4_, 1.5 MgSO_4_, 1 CaCl_2_, 26 NaHCO_3_, and 10 glucose, and had a pH value of 7.4 with 95% O_2_-5% CO_2_. Subsequently, mice were decapitated and the cord was transected at the rostral side of the lumbar enlargement. The transected spinal cord with the attached roots was quickly transferred to a 100 mm Petri dish filled with oxygenated ACSF. Dorsal and ventral roots unrelated to S2–S3 segments were removed, and the cord at the caudal edge of the lumbar enlargement was transected. The cord with S2–S3 ventral roots and the dorsal roots was then transferred to a recording chamber, a 55 mm Petri dish with silicone elastomer (Sylgard) on the bottom, which was filled with oxygenated ACSF. The cord was positioned with its ventral side facing up and pinned at its rostral/caudal ends. Two homemade electrode plates containing three bipolar electrodes were placed on the lateral sides of the cord. The ventral and dorsal roots were mounted on the electrodes and were covered with a mineral oil/petroleum jelly (2:1) mixture. The level of oxygenated ACSF was then adjusted to cover the cord at bath volume between 0.2 and 0.3 ml and was circulated at a rate of 1–3 ml/min.

### Ventral Root Recording and Protocols

The electrodes mounted with dorsal roots were connected to an isolation unit (PSIU6E, Grass Instruments) that was in turn connected to a stimulator (S88, Grass Instruments). The four recording electrodes were individually connected to four differential amplifiers (DAM 50, WPI) with gain at 1000X, high-pass filtering at 300 Hz, and low-pass filtering at 20 kHz. The analog signals were digitized at 20 kHz and acquired through a Dell computer through an interface (Digidata 1322A, Axon Instruments). The compound action potentials (coAPs) were evoked by stimulating one of two dorsal roots and recorded on the ventral roots. A threshold intensity to produce minimum coAPs on ipsilateral ventral roots was first determined by an electrical pulse with 0.2 ms duration and with current intensity between 5 and 20 μA. Afterward, a single electrical pulse and a five electrical pulse train at 50 Hz frequency at intensity of 3X threshold were alternately delivered to the dorsal root every 1 min in the remaining recordings. To produce stable and maximal LLRs, the antagonists, strychnine at 5 μM for glycine receptors and bicuculline at 10 μM for GABAA receptors, were applied routinely in each experiment. An effort was also made to maximize the LLR by activating LTCCs through serotonin receptors by α-5-HT at 5 μM and citalopram at 0.3 μM (Perrier and Hounsgaard, 2003; Li et al., 2007) before switching to CPT. The effects of CPT on root reflexes was then evaluated at three different concentrations of 50, 100, and 200 μM. Nimodipine, a non-selective calcium channel blocker was tested at 50 and 100 μM to compare with the effects of CPT. CPT was a gift from Dr. Richard B. Silverman (Department of Chemistry, Northwestern University). All other drugs were purchased from Sigma-Aldrich.

### Intracellular Recording and Protocols

Intracellular recording was performed to test the effect of CPT on Ca PICs, which are involved in intrinsic firings in response to a depolarizing triangle current in cSCI MNs. Glass pipette electrodes (603000, WPI Instruments, Inc.) were pulled with a Sutter puller (P-97, Sutter Instrument Co.) and filled with 3 M KCl in order to have a resistance of 20–25 MΩ. The electrodes were driven into the ventral horn using a stepper-motor (Model 2660 Micropositioner, David Kopf Instruments). The recorded electrical signal was amplified using an Axoclamp 2A amplifier (Axon Instruments) in the bridge mode and digitized at 20 kHz into a Dell computer through the same interface (Digidata 1322A, Axon Instruments). Upon penetration, neurons were hyperpolarized with a 1–3 nA negative DC current that was gradually withdrawn within 3 min depending on membrane potentials. The intracellular recordings started after the penetrated neurons were identified as MNs by their concurrent firings with one ventral root, or by the AP evoked antidromically on the ventral root. The condition of the MN was monitored by its resting membrane potential, AP and responses to a −0.05 nA current pulse with 250 ms duration. The MNs with resting membrane potentials more negative than −60 mV and overshooting potentials were selected to test CPT. To evoke the intrinsic firing, triangle depolarizing currents up to 7 nA and of 20 s duration (half ascending and half descending in current intensity) were injected into the MN through the glass electrode every 1 min. CPT at 100 μM was applied to the recording solution for up to 60 min after 5 trials of control recordings.

### Data Analysis

To analyze root reflexes, raw data were first rectified and the baseline noise was removed by subtracting the same period of control recording before the stimulation. The value of each root reflex component is the summation of its rectified voltage in its time window, i.e., area under curve (AUC), with unit of mV ms. To evaluate the pharmacological effects of CPT and nimodipine on LLR responses, the absolute LLR values were converted to percentage changes using 10 trials of averaged absolute LLR values before CPT application as control. For intracellular recordings, membrane potentials and resistances, and firings in response to current injections were quantified to evaluate the effects of CPT on intrinsic firings. Statistical significance was analyzed by t test, balanced and unbalanced two way ANOVA tests, *post hoc* t and Tukey tests, and wilcoxon signed rank test (^∗^*p* < 0.05, ^∗∗^*p* < 0.01, and ^∗∗∗^*p* < 0.001).

## Results

A total 56 female adult mice were tested in this study. We first established a protocol for stable measurement of LLRs in both aSCI and cSCI mice. The effects of CPT on LLRs were then evaluated and compared with those from nimodipine and ketamine. With intracellular electrode recordings, we measured neuronal activities of sacral MNs in aSCI and cSCI mice and the effects of CPT on intrinsic firings in those MNs in cSCI mice. In cSCI mice, enhanced muscle activities on hindlimbs could be frequently observed, but not quantified in this study.

### Quantifications of Root Reflexes in SCI Mouse Models

**FIGURE 1:**
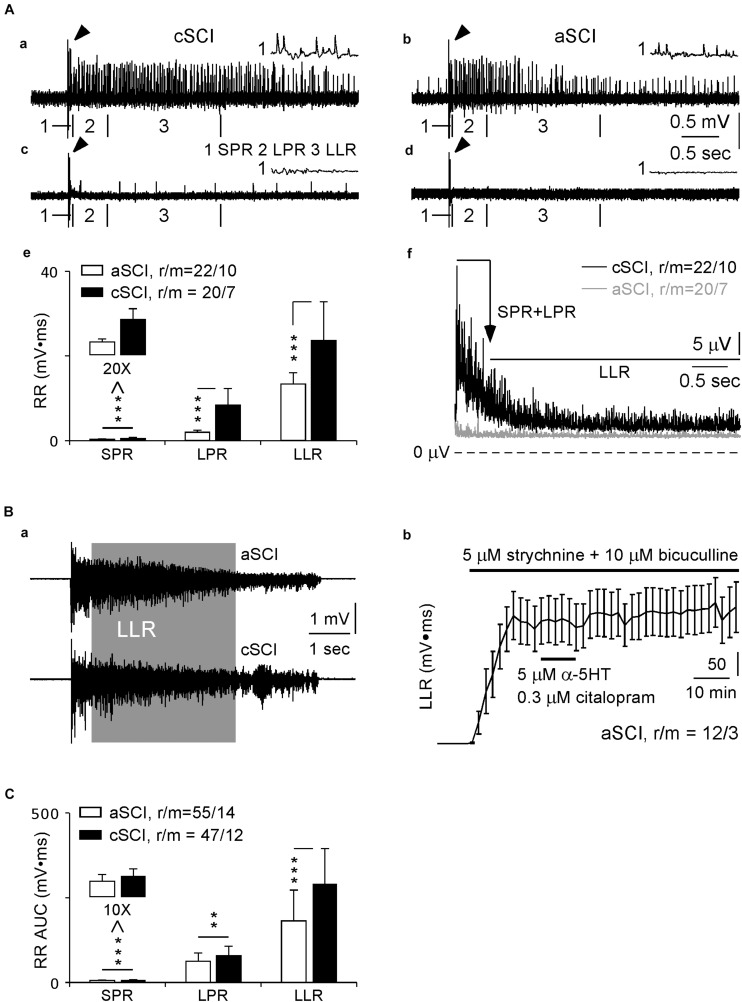
Evoked root reflexes. **(A)** Ventral root recordings without blocking inhibitory synapses. Four sample recordings from 2 cSCI mice (a,c) and 2 aSCI mice (b,d) display the effects of single stimulation at 3X on root reflexes, in which the LLR was evoked in (a–c), but not in (d). The histogram in (e) shows AUC values of rectified root reflexes for each component in aSCI and cSCI mice. The plots in f display rectified and averaged root reflexes in duration of 10–4,000 ms. (1) arrows indicate stimulus artifact; (2) the artifacts and mono-synaptic reflexes are truncated; (3) the insets marked by number (1) show expanded SPR, and number (2–3) with boundary bars indicate the locations of LPR and LLR. **(B)** The root reflexes after blocking inhibitory synapses. Two sample recordings in a display full length of root reflexes from both aSCI and cSCI mice. The plot in (b) shows stable recordings of LLRs from 12 ventral roots in three aSCI mice. **(C)** The histograms compare three components of root reflexes between aSCI and cSCI mice. Significant difference was determined by unbalanced two way ANONA (mouse type: *p* = 1.17 × 10^–7^, components: *p* = 2.45 × 10^–7^) and asterisks represent the significance of *post hoc t*-tests (**p < 0.01, ***p < 0.001). r/m, roots/mice; error bars, SD.

In SCI rats (Murray et al., 2011a,b), a single electrical pulse at 3X threshold is able to activate long-lasting root reflexes containing three cellular components: short-latency polysynaptic reflex (SPR, 10–40 ms), longer polysynaptic reflex (LPR, 40–500 ms), and long-lasting reflex (LLR, 500–4,000 ms) which involves activation of Ca PIC (Murray et al., 2011a,b). Using this protocol, we recorded root reflexes in 20 ventral roots from 7 cSCI mice that displayed all the three components with varied intensities (Figures 1Aa,c). In contrast, the evoked root reflexes were much weaker in aSCI mice (21 ventral roots in 10 mice, Figure 1Ad) except clear LLRs were only observed in 1 ventral roots in 1 aSCI mouse (Figure 1Ab). Statistical analysis of their AUC values indicated significant enhancements of the root reflexes for all three components in cSCI mice (Figure 1Ae), suggesting hyperexcitability in their spinal motor system. The long lasting feature of the LLR was also confirmed in cSCI mice by the time course of the averaged-rectified root reflexes (Figure 1Af).

Due to the small values of LLRs in the above experiments, Ca PICs underlying the LLR are likely activated only weakly. To explore detailed pharmacological influence of CPT on LLRs, stronger activation of Ca PICs would be necessary. We therefore tested the effects of blocking inhibitory synaptic transmission with strychnine, an antagonist of glycine receptors, and bicuculline, an antagonist of GABAA receptors, and enhancing Ca PICs with α-5-HT and citalopram, a selective 5-HT reuptake inhibitor, as shown by Li et al. (2007). When 5 μM strychnine, 10 μM bicuculline, 5 μM α-5-HT and 0.3 μM citalopram were added to the recording solution, maximum LLRs could be reached rapidly in both types of SCI mice, shown in Figure 1Ba as examples. After withdrawal of α-5-HT and citalopram at 10 min of the treatment, the stable and prolonged recordings were confirmed in three aSCI mice (Figure 1Bb), which could be the control to the effects of CPT as the drug had slow recovery especially at 200 μM. In addition, for the recordings at the end of application of α-5-HT and citalopram, we compared the magnitudes of LLRs induced by single and five pulse stimulations and did not find significant differences (single pulse: 319.61 ± 169.77; five pulse: 324.45 ± 174.08; mean AUC ± SD; roots/mice: 33/9 from 18/5 aSCI mice, 15/4 cSCI mice; paired *t*-test, *p* = 0.6132), suggesting maximum activation of LLRs. It was noticeable that during the recordings, spontaneous bursting occurred occasionally due to the cocktail treatment. However, its impact on the evoked LLRs was very limited when the stimulation interval was set at 1 min. This protocol was then used in all remaining root reflex experiments.

With this cocktail protocol, we first quantified the evoked root reflexes between aSCI and cSCI mice, and found that they had similar durations (aSCI mice: 8.41 ± 2.95, 55/14; cSCI mice: 9.16 ± 2.27, 47/12; *p* = 0.1466. mean seconds ± SD, roots/mice, *t*-test), but magnitudes of all components, i.e., SPR, LPR, and LLR, were significantly enhanced in the cSCI mice compared to the aSCI mice (Figure 1C), suggesting the enhancement of maximum excitability in the spinal motor system in cSCI mice.

### Effects of CPT on LLRs

**FIGURE 2:**
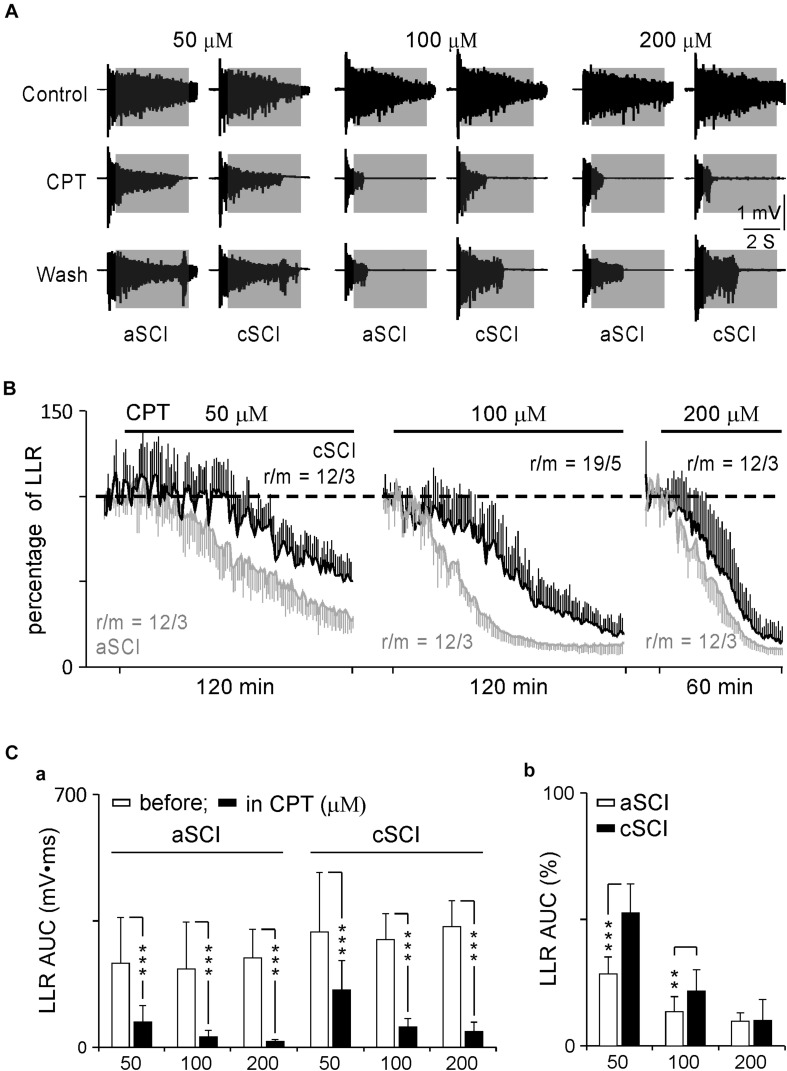
Effects of CPT on LLRs. **(A)** Sample recordings display the inhibitory effects of CPT on LLRs at different concentrations. Shadow areas mark LLRs. **(B)** Time courses of inhibitory effects of CPT on LLRs. The AUC values of LLRs are converted to percentage changes using the controls before the drug’s application. r/m, roots/mice. **(C)** Histograms display the inhibitory effects of CPT on LLRs. The histograms in a shows AUC values before and after CPT applications at three concentrations for both types of mice. The statistical significance is determined by paired *t*-test and indicated by asterisks (***p < 0.001). The histograms in b compare the percentage changes of LLRs between aSCI and cCSI mice. The significant differences are analyzed by unbalanced two way ANOVA (mouse type: *p* = 6.11 × 10^–10^, CPT concentration: *p* = 2.03 × 10^–23^) and *post hoc t*-test (**p < 0.01, ***p < 0.001).

**FIGURE 3:**
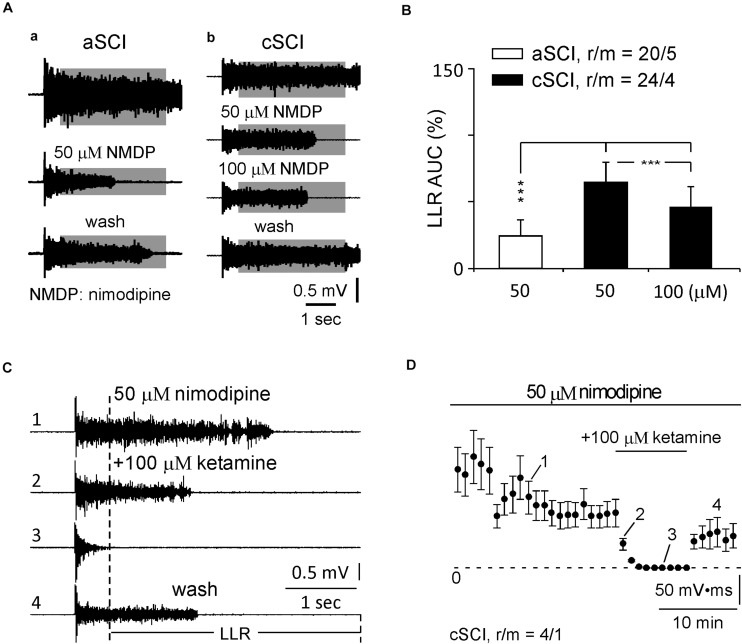
Inhibition of nimodipine and ketamine on LLRs. **(A)** Sample recordings show the responses of LLRs to nimodipine at 50 and 100 μM in an aSCI and a cSCI mouse. **(B)** Histogram shows the statistics of nimodipine’s effects on LLRs. r/m, roots/mice. **(C)** Sample recordings of LLRs in nimodipine and ketamine. Numbers (1–4) indicate the recordings before, in and after ketamine treatment. **(D)** Time course of LLRs in nimodipine and ketamine. The plot displays the averaged AUC values of LLRs with numbers (1–4) matching numbers (1–4) in **(C)**. The asterisks represent the results of *t*-tests (***p < 0.001), and r/m represents roots/mice.

CPT is now identified as an inhibitor of Ca_V_1.3 channels with a > 600-fold potency over Ca_V_1.2 channels (Kang et al., 2012; Cooper et al., 2020). It may provide a safer therapy on SCI-induced spasms considering the distributions of Ca_V_1.2 and Ca_V_1.3 in the nervous system versus the cardiovascular system (Lipscombe et al., 2004; Catterall et al., 2005; Striessnig et al., 2006), and lack of selective antagonists on LTCCs (Xu and Lipscombe, 2001). To test this potential, we started with low concentrations of CPT (10–20 μM) during a 30 min application, which produced a weak inhibition, if any, on the LLRs (data not shown). We then tested CPT at three increased concentrations (50, 100, 200 μM) over a prolonged duration (120 min for 50–100 μM and 60 min for 200 μM). These changes resulted in clear inhibitory effects on the LLRs (Figure 2). Statistical analysis showed that CPT significantly inhibited the LLRs in each group in both types of mice after its application (Figure 2Ca) while it produced stronger inhibition on the LLRs for the aSCI mice than for the cSCI mice (Figure 2Cb). The fact that CPT at 50–100 μM, but not at 200 μM, had weaker inhibition on LLRs in the cSCI mice than in the aSCI mice (Figure 2Cb) suggests either component changes of Ca_V_1.3 channels (Huang et al., 2014) or up-regulation of other cellular component(s) in the cSCI mice. Meanwhile, the inhibition of CPT at 200 μM might become non-selective as it almost equally inhibited the LLRs in both mouse groups. To address these possibilities, we compared the inhibitory effect of nimodipine on the LLRs between the two mouse groups. As nimodipine at 20 μM had been reported to be able to completely block Ca PICs (Li and Bennett, 2003; Li et al., 2004), we applied nimodipine at 50 μM to aSCI mice and 50–100 μM to cSCI mice and assumed a complete blockage of Ca PICs. Under these conditions, significantly bigger LLRs were still recorded in the cSCI mice at 50 μM and even at 100 μM than in the aSCI mice at 50 μM (Figures 3A,B), which supports the ideas that component changes of Ca_V_1.3 and up-regulation of other cellular components are indeed occurring. That CPT at 200 μM produced stronger inhibition of the LLRs in the cSCI mice than nimodipine at 100 μM suggests that at high enough concentrations, it becomes non-selective (nimodipine for cSCI mice: 47.74 ± 14.16, r/m = 24/4; CPT for cSCI mice: 13.23 ± 6.21, r/m = 12/3; *p* = 8.86 × 10^–12^, mean% ± SD).

To further test possible involvement of other cellular components, we measured the effect of blocking NMDA receptors on the LLRs in the cSCI mice as the density of these receptors were found to be enhanced in an SCI rat model (Tu et al., 2017). Among several antagonists, ketamine, a non-competitive antagonist of NMDA receptors (Anis et al., 1983), was selected because it produces moderate inhibition on NMDA receptors (Kang et al., 2017), which may benefit the design for *in vivo* test. When applied at 100 μM following the treatment of nimodipine in 4 ventral roots in 1 cSCI mouse, ketamine quickly blocked the LLRs, indicated by 2,3 in Figure 3C as examples and plotted in Figure 3D to show the time course of averaged LLRs with steady recovery, supporting the contribution of NMDA receptors.

**FIGURE 4:**
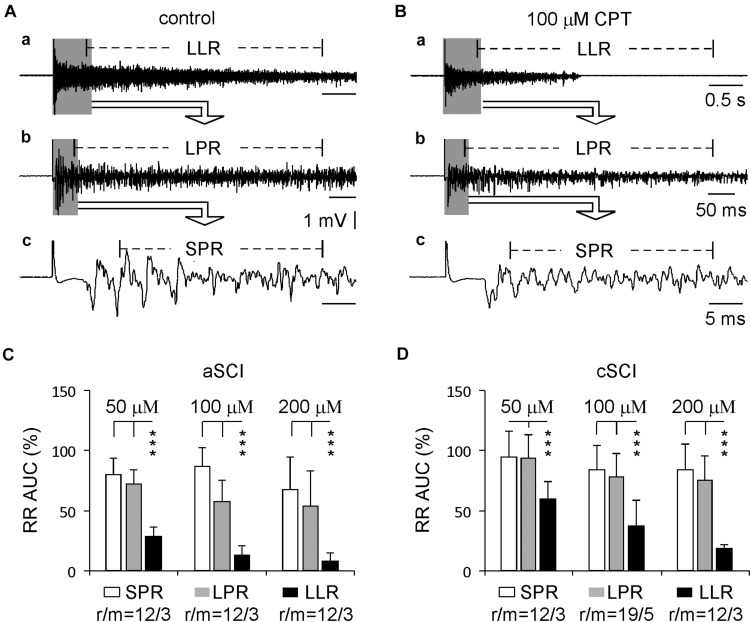
Comparison between different components of root reflexes. **(A)** Sample recordings before CPT application in a cSCI mouse display root reflexes with different components: LLR (a), LPR (b), and SPR (c). **(B)** Sample recordings from the same mouse show the inhibitory effect of CPT on these root reflexes (a–c). **(C)** Histograms illustrate the inhibitory effects of CPT on the root reflexes in aSCI mice. The data were analyzed by unbalanced two-way ANONA (drug concentrations: *p* = 0.0040; components of root reflexes: *p* = 4.72 × 10^–25^) and by *post hoc* Tukey test (***p < 0.001). **(D)** Histograms display CPT’s effects on the root reflexes in cSCI mice. The data were analyzed with the same statistics as in **(C)** (drug concentrations: *p* = 3.58 × 10^–6^; components of root reflexes: *p* = 4.38 × 10^–23^; unbalanced two way ANOVA; *post hoc* Tukey test: ***p < 0.001). r/m represents root/mouse; the data were collected in the same experiments as used in Figure 2.

Previous reports have shown that the spasms in SCI rats are mainly due to the enhancement of the LLR, but not the SPR or LPR (Murray et al., 2011a). Here, we compared the percentage inhibition of CPT on all three components of the evoked root reflexes from the same experiments testing CPT on the LLRs. The results (Figure 4) show that at three concentrations, both SPRs and LPRs were significantly less inhibited by CPT, confirming the major role of Ca_V_1.3 in conducting LLRs in both aSCI and cSCI mice.

### Intrinsic Properties of MNs and the Effects of CPT

**FIGURE 5:**
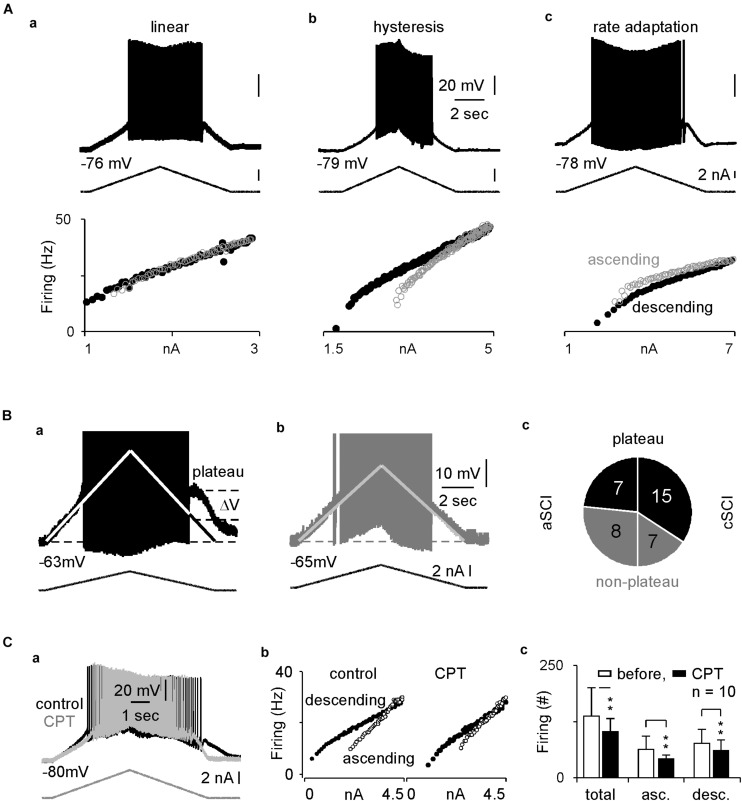
Intracellular recordings and inhibitory effects of CPT on MNs. **(A)** Sample recordings show three types of intrinsic firings in (a–c). The top, middle and bottom rows display membrane potentials, ramp current and scattered frequency-current relations. **(B)** Plateau potentials. The sample recordings show plateau potential as indicated in a MN from a cSCI mouse in (a), but not a MN from an aSCI mouse in (b). The pi plot in c indicates distribution of plateau potentials in MNs between aSCI and cSCI mice. (1) the white lines represent linear relation between membrane potential and current injection due to passive membrane resistance, upon which the amplitude of plateau potential is estimated; (2) APs are truncated to have better resolution to display plateau potential. **(C)** The effect of CPT on intrinsic firing. Two overlapped sample recordings show intrinsic firings before and after CPT application in a MN from a cSCI mouse in (a), which is converted to scattered frequency-current plots in (b). Histograms in c illustrate the inhibitory effect of CPT on intrinsic firings during wholecurrent injection and different phases of current injection. The asterisks represent the result of *t*-tests (**p < 0.01).

In rat SCI models, enhanced firings and plateau potentials are characteristics of the spinal MNs, which underlie the LLRs and lead to muscle spasms (Murray et al., 2011b). LTCCs are known to play an important role in these intrinsic properties (Li and Bennett, 2003). To determine the relative contribution of Ca_V_1.3 channels in the LLRs in spinal MNs in our cSCI mice, we measured the intrinsic properties and the effect of CPT on these SCI MNs. In 37 recorded MNs (Table 1A), we found that the resting membrane potentials and firing thresholds are similar between aSCI and cSCI MNs, but the membrane resistances are significantly bigger in the cSCI MNs than in the aSCI MNs, suggesting that the cSCI MNs are more sensitive to synaptic inputs. We then applied ramp depolarizing currents to measure intrinsic firings. The results show that induced firings could be categorized into 2 major firing patterns as linear (Figure 5Aa) and hysteresis (Figure 5Ab) with only 1 MN showing rate adaption (Figure 5Ac). Chi square analysis indicates that significantly more cSCI MNs than aSCI MNs fired with hysteresis (Table 1B), a typical firing pattern in cSCI MNs (Bennett et al., 2001). In addition, the plateau potential (Figure 5Ba), an important determinant for sustained firing, was observed more frequently in the cSCI MNs than in the aSCI MNs (Figure 5Bc, *p* = 0.0368, chi-square). Taken together, these data suggest an enhancement in intrinsic excitability in these cSCI MNs.

**TABLE 1:** Basic membrane properties and firing types.

**A**								
	**RMP (mV)**		**Threshold (mV)**			**MR (MΩ)**		
			
Group	aSCI	cSCI	aSCI		cSCI	aSCI		cSCI
Mean	−73.44	−71.35	−49.98		−49.97	5.04		7.81
*SD*	±6.26	±8.35	±4.18		±5.35	±1.73		±2.66
*N*	15	22	15		22	15		22
*T*-test	p = 0.3820		*p* = 0.9962			*p* = 0.0009		

**B**								

		**L**		**H**			**RA**	

n (aSCI)		10		5			0	
n (cSCI)		11		10			1	
Chi square test		*p* = 0.0390						

**RMP, resting membrane potential (mV); MR, membrane resistance; L, linear; H, hysteresis; RA, rate adaption.**

To test the effect of CPT on Ca_V_1.3 channels, we measured the triangle depolarizing current induced firings in 10 cSCI MNs. After the first few recordings, CPT at 100 μM was added to the ACSF, and the recording was continued for up to 30 min with one recording every 1 min. The results showed that CPT significantly enhanced firing threshold without clearly affecting resting potential and membrane resistance (Table 2). Accordingly, CPT significantly reduced the intrinsic firings in both ascending and descending phases during the injection of triangle depolarizing currents, which is shown in Figure 5Ca as an example with its scattered frequency-current plots in Figure 5Cb, and plotted in Figure 5Cc. Taken together, these data support a therapeutic potential of CPT in SCI induced spasms through inhibiting Ca_V_1.3 channels and reducing LLRs.

**TABLE 2:** Effects of CPT on membrane properties.

	RMP (mV)		Threshold (mV)		MR(MΩ)	
			
CPT	Before	After	Before	After	Before	After
Mean	−71.74	−71.59	−49.33	−46.31	7.95	8.21
*SD*	7.91	9.24	5.59	7.39	2.81	2.25
*N*	10		10		10	
WSRT	*p* > 0.05		*p* < 0.05		*p* > 0.05	

**WSRT, wilcoxon signed rank test; CPT concentration = 100 μM.**

## Discussion

In this study, we measured root reflexes and intrinsic firings of spinal MNs in sacral spinal cords in two SCI mouse models. We evaluated the therapeutic potential of CPT on these neuronal activities. Our results suggest an enhanced excitability of the spinal motor system in cSCI mice and the involvement of Ca_V_1.3 channel in the excitability.

### SCI and Spasms

In humans, SCI most commonly occurs within the cervical segment of the spinal cord. The frequency of SCI then decreases rostrally along the spinal cord with less in the thoracic and lumbar segments and the least in the sacral spinal cord (Tauqir et al., 2007; van den Berg et al., 2010; Thompson et al., 2015). After the initial period of SCI, most individuals (65–78%) develop muscular spasms with a higher rate for rostral SCI and a lower rate for caudal SCI (Maynard et al., 1990), and the severity of spasm is likely to depend on both severity and location of injury. Thus, nuance may exist in the mechanisms of spasm and may affect its therapy. In order to study the various mechanisms underlying spasms and to develop more efficient therapies, several animal models have been developed with different emphases and advantages. In an SCI rat model with the spinal cord completely transected at the second sacral segment (S2), severe spasms occur in 3 weeks (Bennett et al., 1999). When completely transected at the 9–10th thoracic segments, longer times (2–3 months) are needed for clear spasms to develop (Skinner et al., 1996; Corleto et al., 2015), suggesting segmental differences in neuronal processing after SCI. Such differences appear to exist as well in SCI mouse models. In an SCI mouse model, spasms could be observed as early as in 6 weeks after complete transection of the spinal cord at the S2 level (Lin et al., 2019). Thus, the developmental profile of SCI-induced spasms could be an important indicator of pathophysiological process underlying SCI and would be helpful in determining appropriate therapy, which however needs to be validated further by systematic study.

Spasms cause increased muscle tone and results directly from LLRs in the spinal motor system. Therefore, to evaluate a drug for its therapeutic potential, LLRs would be an important parameter with which one can observe the drug’s effect. However, our data suggest that the LLR induced under intact conditions is only a small portion of its maximum amplitude and it may not be the best choice for pharmacological study. By blocking inhibitory synaptic transmissions and activating LTCCs through serotonin receptors (Li et al., 2007), the LLRs can be fully expressed, and are demonstrated to be hyperactive and to contain multiple cellular components in SCI mice. These *in vitro* results will likely benefit the design for further *in vivo* test. Thus, we have validated a useful approach for scanning for potential anti-spasm medications.

### Cellular Mechanisms Underlying LLRs

CPT is a synthetic chemical developed as a selective inhibitor against Ca_V_1.3 channels for therapy in Parkinson’s disease (Kang et al., 2012, 2013), which is now identified as a selective negative allosteric modulator of Cav1.3 by binding to dihydropyridine (DHP) binding pocket on CaV1.3 subunits (Cooper et al., 2020). CPT was first reported to be very potent and selective (Kang et al., 2012), but was later found to be modest against Ca_V_1.3 channels (Huang et al., 2014). This variation of CPT is due to the existence of splice variants of Ca_V_1.3 channels, which have various biophysical and pharmacological properties (Bock et al., 2011; Tan et al., 2011; Huang et al., 2013). The expression of various Ca_V_1.3 splice variants could be tissue-selective (Verma and Ravindranath, 2019). Thus, the effective concentration of CPT at 50–100 μM used in our study may reflect the presence of specific splice variants in the spinal motor system. Our study also suggests a possibility that the pathophysiological processes of SCI may change the splice variants of Ca_V_1.3 channels as at the same concentration, CPT has weaker inhibition on LLRs in cSCI mice. Another possibility is that CPT’s differential effects observed between aSCI and cSCI mice may result from up-regulation of NMDARs. In an SCI rat model, NMDARs’ subunits, NR1 and NR2A, at both protein and mRNA levels, were found to be over-expressed in the ventral horn at the caudal end of the injured cord (Tu et al., 2017), indicating a possible involvement of NMDA receptors in LLRs. Indeed, our data showed that the LLRs could be inhibited by blocking NMDARs. As the only currently available inhibitor for the Ca_V_1.3 channel, CPT does display therapeutic potential for SCI-induced spasms. However, a combination therapy may be more efficient as our data showed multiple pathophysiologies involved in SCI.

Recently, nimodipine was reported to prevent the development of spasms through early and prolonged application in SCI mice. Its inhibitory effect against hyperexcitablity on calcium influx is also under investigation for other neurological diseases such as Parkinson’s and multiple sclerosis (Guzman et al., 2010; Ingwersen et al., 2018). However, nimodipine is a non-selective L-type calcium channel blocker with higher potency for Ca_V_1.2 than Ca_V_1.3 (Park et al., 2015). Thus, its side effects on the cardiovascular system may diminish its therapeutic application (Kang et al., 2012). As we showed the inhibitory effect of CPT on LLRs and Ca_V_1.3-related intrinsic firing, it would be interesting to examine whether CPT could produce a similar effect on preventing the development of spasms after SCI.

In summary, spasm development in SCI is due to enhanced excitability in the motor system through multiple cellular mechanisms. Our results in this study suggest a role for Cav1.3 channels in the enhanced excitability and the potential of CPT as a novel therapy for SCI-induced spasms. Ca_V_1.3 channels are likely involved in several neurodegenerative disease and therefore further study of CPT may lead to multiple therapeutic applications.

## Data Availability Statement

The raw data supporting the conclusion of this article will be made available upon request.

## Ethics Statement

The animal study was reviewed and approved by the University Animal Research Committee.

## Author Contributions

MJ, CH, and VT interpreted data. MJ, DB, CH, and VT prepared the manuscript. All authors reviewed and approved the manuscript.

## Conflict of Interest

The authors declare that the research was conducted in the absence of any commercial or financial relationships that could be construed as a potential conflict of interest.
